# Sex and age effects in past-year experiences of violence amongst adolescents in five countries

**DOI:** 10.1371/journal.pone.0219073

**Published:** 2019-07-08

**Authors:** Lindsay Stark, Ilana Seff, Anna Hoover, Rebecca Gordon, Daniela Ligiero, Greta Massetti

**Affiliations:** 1 George Warren Brown School, Washington University in St. Louis, St. Louis, Missouri, United States of America; 2 Department of Population and Family Health, Columbia University Mailman School of Public Health, New York, New York, United States of America; 3 Together for Girls, Washington D.C., United States of America; 4 Centers for Disease Control and Prevention, Atlanta, Georgia, United States of America; Stellenbosch University, SOUTH AFRICA

## Abstract

**Purpose:**

To date, there has been insufficient focus on age and sex differences in studies of violence amongst adolescents and young adults in low- and middle-income countries. As adolescence is a formative period during which experiencing violence can have both short- and long-term consequences, we aim to investigate experiences of violence by age and sex across five countries.

**Methods:**

Incidences of past-year violence victimization were estimated by sex across two-year age bands (13–24 years) using Violence Against Children Survey datasets from Cambodia, Haiti, Kenya, Malawi, and Tanzania. Analyses were conducted separately for each country. The presence of an association with age and each type of violence was identified using logistic regressions separately by sex. Sex was then added to the models as an interaction term and adjusted Wald tests were used to assess differences between males and females in age effects.

**Results:**

Risk of physical violence by both an adult caregiver and a community member decreased with age for both sexes in all countries. In contrast, risk of IPV increased with age for both sexes in all countries. Although some countries displayed a steeper increase in risk of IPV and sexual violence with age for males, females face higher overall levels of risk for these forms of violence.

**Conclusion:**

Findings highlight how adolescents’ and young adults’ risk of violence changes with age and type of violence. The analysis underscores the importance of collecting violence data disaggregated by age and sex to best inform policies and programming.

**Implications and contributions:**

We analyzed five Violence Against Children Surveys (VACS) and found age effects for physical, sexual, and intimate partner violence for adolescents 13–24 years old. Age effects for sexual violence are stronger among females than males. Future policies targeting adolescents should consider how age and gender influence risk of violence.

## Introduction

Adolescents and young adults, defined as individuals 10–24 years of age, account for 24% of the world’s population, or approximately 1.8 billion people [1,2]. Adolescence is both a formative period of development in the life course, as individuals transition from childhood to adulthood, and a period characterized by heightened vulnerabilities to certain risks that can have a substantial impact on future health trajectories [3]. Gender and sexuality take on increased importance, and vulnerability to a number of forms of violence increases, with short and long-term consequences [4–6]. Additionally, as children enter adolescence, inequitable gender norms become increasingly embedded, which in turn contributes to increased exposure—defined for the purposes of this study as victimization—to violence [7]. Due in part to these social factors and the rapid biological changes that characterize this period, females and males have very different experiences through adolescence and into young adulthood [8].

Meanwhile, violence in childhood and adolescence is increasingly recognized as common, and a growing body of evidence underscores the association of this violence with a constellation of short- and long-term health, well-being, and behavioral consequences. Empirical findings suggest that experiencing and witnessing violence during adolescence are both associated with the development of psychiatric sequelae, including Posttraumatic Stress Disorder, depressive disorders, Generalized Anxiety Disorder, and suicidal ideation [9–11]. Past-year exposure to violence in adolescents has been found to be predictive of cortisol reactivity in the subsequent 12 months, indicating elevated stress [11]. Experiences of physical, sexual, and psychological abuse during childhood and adolescence can have significant negative effects on the lifelong burden of illness and disease, increasing risk of addiction, obesity, and other chronic illnesses [12]. Adolescent girls and young women who experience sexual violence have higher rates of unintended pregnancy, HIV, and other sexually transmitted infections [13,14]. Additionally, male survivors of sexual violence have a high risk of experiencing adverse mental health outcomes, substance abuse, exposure to HIV and STIs, and harmful gender attitudes, which potentially lead to males becoming perpetrators themselves [15, 16].

Despite growing recognition of the importance of investigating risk factors for violence and its outcomes using a life course approach, a few limitations remain in the way these topics have been studied. There is a dearth of literature related to the epidemiology and effective interventions in low and middle-income countries (LMICs) [17, 18, 19]. Insufficient attention has been given to investigating risk factors, including sex and age differences, despite the fact that, child maltreatment occurs at much higher rates and creates more systemic burdens in LMICs than high-income countries [20]. There has been a tendency to view adolescence as a monolith, assessing types of violence such as corporal punishment without examining the potential impact of gender or gender disparities [21]. At the same time, much of the research on violence against adolescents globally highlights the experiences of females and their elevated risks of sexual violence [22, 23]. While this emphasis is important, the narrow framing has failed to provide a full gender analysis of the ways in which violence affects *both* males and females as they grow and develop. These types of analyses can help inform unique policy and program needs that may be warranted to address differences in experiences and outcomes for males and females.

While some studies have considered gendered experiences of violence during adolescence and young adulthood, very few have taken a cross-national approach [7]. Further, these studies also fail to look at a wide range of ages, or fail to look at experiences across narrow age bands within a wider range of ages; traditional analyses look at violence during childhood or adulthood as a whole, or in larger age groupings (for example, 10–19 years). These analyses provide important overarching information on violent experiences, but limited insight into the sex- and age-specific nuances around development, experience, risk, protection, and impact.

This paper examines past-year experiences of violence for 13–24 year olds, assessing how these exposures differ across age and sex in order to better understand how different typologies of violence impact males and females across adolescence and into young adulthood, and to offer a more nuanced understanding of violence exposure as children move into adulthood. The present study assessed exposure to past-year violence for females and males ages 13–24, across two-year age bands. Data from the Violence Against Children Surveys (VACS) from five countries were used. VACS are led by the U.S. Centers for Disease Control and Prevention (CDC) as part of the Together for Girls (TfG) public-private partnership (www.togetherforgirls.org) [24]. VACS are nationally representative surveys of youth that use comparable questions and standard methodology across countries, in order to: (1) estimate the national prevalence of sexual violence, physical violence, and emotional violence against males and females; (2) identify risk and protective factors; (3) identify health and well-being consequences; (4) assess disclosure of violence, knowledge and utilization of services, as well as barriers to accessing services; and (5) identify areas for further research. Surveys also include questions on demographic characteristics, sexual experiences, and knowledge and attitudes towards HIV and HIV testing, among many others. The purpose of the present study was to assess whether there are age effects for different types of violence in adolescence and young adulthood, stratified by sex. To our knowledge, this is the first such analysis using multi-country study data on violence against adolescents and young adults.

## Methods

The VACS public use datasets from Malawi (conducted in 2013), Kenya (2010), Cambodia (2013), Haiti (2012), and Tanzania (2009) were used in the present analyses [25, 26]. Response rates for all countries were high: Malawi, females 84·4%, males 83·4%; Kenya, females 84·8%, males 80·4%; Cambodia, females 91·0%, males 89·9%; Haiti, females 85·6%, males 82·0%; Tanzania, females 93·3%, males 92·5%. VACS utilize a multi-stage cluster sample design, employing a “split sample” approach such that each enumeration area (EA) is either all-male or all-female. This approach minimizes the risk of interviewing a survivor and perpetrator in the same EA. The VACS adheres to World Health Organization guidelines on the safety and ethics in studies of violence against women, and in accordance with the guidelines, frames the survey as a study on health and life experiences. After obtaining caregiver consent, trained interviewers obtain informed assent from respondents younger than 18. To further protect confidentiality, consent and assent procedures and interviews are conducted in private spaces in and around the homes of respondents. The VACS do not collect any personally identifying data. The protocols for the surveys in each country were approved by the CDC as well as in-country Institutional Review Boards [27].

### Outcomes of interest

We estimated the incidence of self-reported exposure to various forms of violence in the last 12 months across two-year age bands for males and females in each country. We included violence types available in all five countries: physical violence perpetrated by a parent or adult caregiver, physical violence perpetrated by an adult in the community, physical or sexual intimate partner violence (IPV), and sexual violence (by any perpetrator). Questions on IPV are not included in Haiti’s questionnaire, so no estimates for IPV are presented for that country. Exposure to each type of violence is operationalized as a dichotomous variable signifying whether the respondent experienced each type of violence within the last 12 months. Specific questions used to construct these dichotomous variables vary slightly by country and can be found in each country’s VACS questionnaire (available upon request).

### Analysis

Analyses were conducted separately for each country in the sample. We first estimated the incidence of each type of violence for males and females, separately. Differences between sexes were assessed using adjusted Wald tests. We then estimated the incidence of each type of violence for males and females, separately, across six two-year age bands: 13–14, 15–16, 17–18, 19–20, 21–22, and 23–24 years old. Two-year age bands were used to ensure age effects were as specific as possible and sample sizes were large enough to be sufficiently powered.

We assessed whether there are age effects for each type of violence exposure for males and females. A variable containing information on each individual’s two-year age-band was included as a continuous effect in a logistic regression estimating violence exposure in the last year. After testing for age effects for males and females, separately, we added sex to the model as an interaction term. Sex differences in age effects were assessed using adjusted Wald tests. All observations were weighted to be reflective of the larger population for each country and standard errors are adjusted for clustering and stratification in the sampling design. All analyses were conducted using Stata14.

## Results

Table 1 summarizes incidence of each type of violence for males and females in all five countries. While the overall likelihood of experiencing each type of violence varies by country, there appear to be consistent differences in exposure to certain types of violence for males and females in multiple countries. Specifically, 13–24 year-old females in all five countries are significantly more likely than their male counterparts to report having experienced sexual violence in the last 12 months.

**Table 1:** Overall sex differences for past-year exposure to violence, all 13–24 year olds.

	Cambodia		Haiti		Kenya		Malawi		Tanzania	
	Males	Females	Males	Females	Males	Females	Males	Females	Males	Females
	No. (%)	No. (%)	No. (%)	No. (%)	No. (%)	No. (%)	No. (%)	No. (%)	No. (%)	No. (%)
Physical violence by parent or other caregiver	61 (4.9)	62 (5.6)	264 (18.1)	319 (21.9)	141 (9.7)	107 (8.7)	182 (16.1)	113 (11.0)*	278 (15.7)	234 (11.9)
Physical violence by adult in the community	64 (5.1)	49 (4.4)	158 (10.8)	149 (10.2)	448 (30.5)	274 (22.3)**	231 (20.4)	124 (12.1)***	483 (27.3)	449 (22.8)
Intimate partner violence	10 (0.8)	30 (2.7)**	---	---	96 (6.6)	107 (8.7)	20 (1.8)	130 (12.6)***	152 (8.6)	165 (8.4)
Sexual violence, any perpetrator	10 (0.8)	39 (3.5)***	(233 (16.0)	337 (23.1)**	106 (7.3)	136 (11.1)*	138 (12.2)	185 (18.0)**	159 (9.0)	291 (14.8)**
Sample size (n)	1,255	1,121	1,459	1,457	1,456	1,227	1.131	1.028	1,771	1,968

Note: Observations are weighted to be representative of the population of 13–24 year-old males and females in each country. Differences in age trends between males and females are assessed using an adjusted Wald test and are adjusted for complex sampling design.

Differences are significant at *p<0.05, **p<0.01, p<0.001.

**Fig 1:**
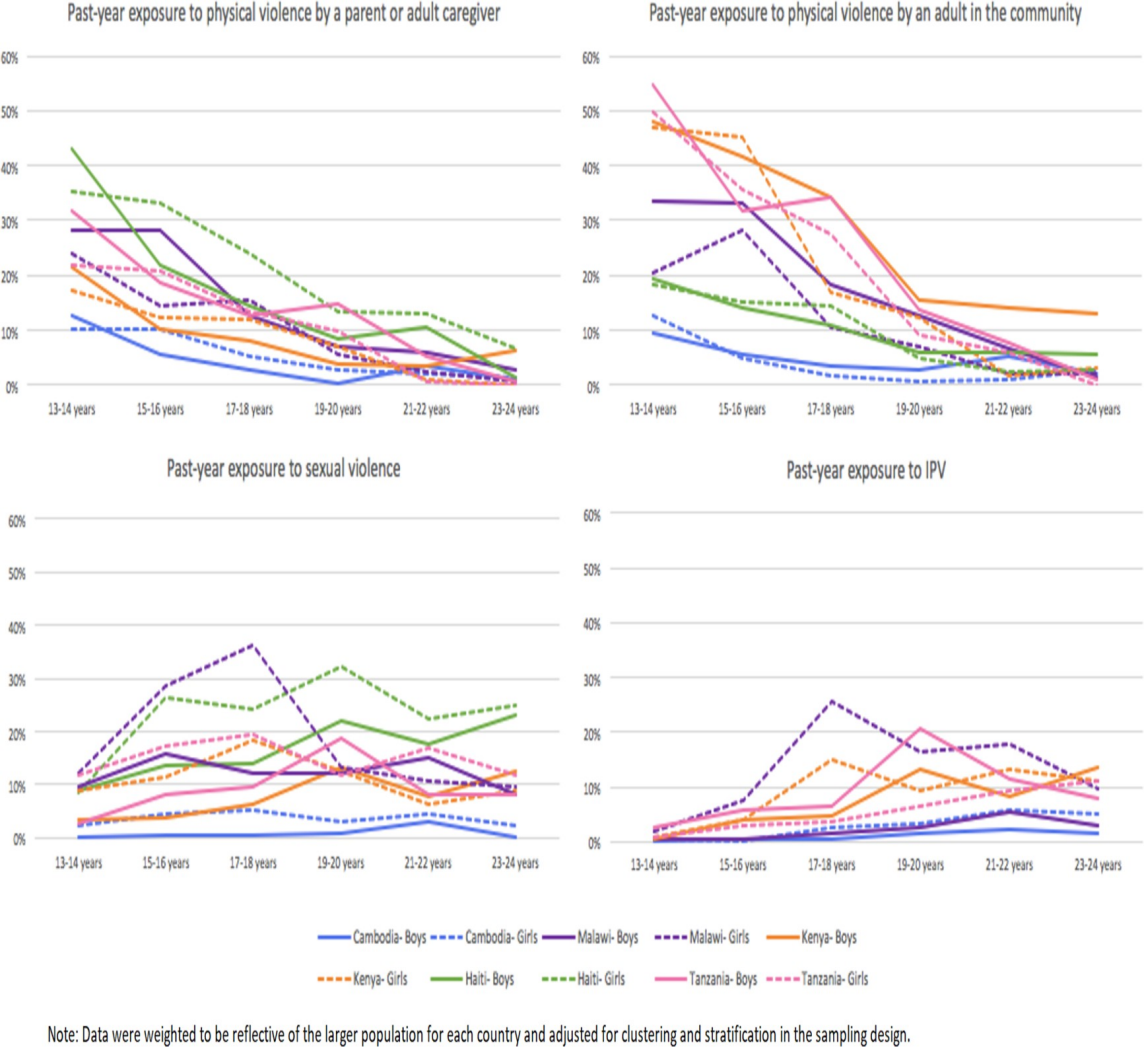
Physical and sexual violence experiences in the past year by age and gender in Cambodia, Haiti, Kenya, Malawi, and Nigeria.

Fig 1 highlights overall age effects for the four types of violence, revealing changes in risk at different ages throughout the 13-24-year-old age group. While past-year exposure to physical violence by a caregiver or adult in the community declined with age across all countries, similar downward effects were not observed for sexual violence or IPV. In contrast, risk of IPV increased with age for males and females in all countries.

### Physical violence by an adult caregiver

Regression analysis revealed that males and females were less likely to experience physical violence by caregivers as they aged in all five countries (see Table 2). While this pattern was consistent across all contexts studied, the relative decline in risk by age differed by sex in Haiti. In Haiti, while results indicated that risk of physical violence by a parent or other caregiver declined for both males and females as they aged, this decline in risk was stronger for males (F = 5·21, p<0·05).

**Table 2:** Sex and age effects for past-year exposure to violence.

	Cambodia		Haiti		Kenya		Malawi		Tanzania	
	Males	Females	Males	Females	Males	Females	Males	Females	Males	Females
	No. (%)	No. (%)	No. (%)	No. (%)	No. (%)	No. (%)	No. (%)	No. (%)	No. (%)	No. (%)
**Physical violence by parent or other caregiver**	** **			** **	** **					
13–14 years	38 (12·5)	26 (10·2)	153 (42·9)	88 (35·2)	71 (21·4)	43 (17·1)	80 (28·1)	53 (24·1)	117 (31·9)	85 (21·7)
15–16 years	14 (5·6)	18 (10·3)	56 (21·9)	90 (33·0)	30 (10·2)	25 (12·2)	65 (28·2)	25 (14·5)	69 (18·5)	70 (20·9)
17–18 years	6 (2·8)	10 (5·4)	41 (14·5)	62 (23·9)	22 (8·0)	25 (12·1)	26 (12·7)	23 (15·6)	44 (12·5)	51 (13·3)
19–20 years	1 (0·4)	4 (2·6)	20 (8·3)	33 (13·5)	7 (0·0)	13 (7·0)	12 (6·9)	9 (5·7)	48 (14·8)	34 (10·0)
21–22 years	6 (3·4)	3 (2·1)	19 (10·5)	30 (12·9)	6 (0·0)	2 (0·9)	7 (5·9)	4 (2·5)	10 (5·1)	2 (0·8)
23–24 years	1 (0·9)	3 (1·3)	2 (1·4)	13 (6·7)	11 (0·1)	0 (0·0)	3 (2·7)	1 (0·7)	1 (0·5)	0 (0·0)
Age effect, OR	0·560***	0·642***	0·547***	0·675***	0·650***	0·588***	0·586***	0·562***	0·599***	0·580***
Interaction between gender and age effect, F-stat	0·63		5·21*		0·56		0·08		0·06	
**Physical violence by adult in the community**										
13–14 years	29 (9·5)	32 (12·7)	69 (19·3)	46 (18·3)	159 (48·0)	120 (47·1)	95 (33·6)	44 (20·3)	201 (54·8)	196 (49·8)
15–16 years	14 (5·5)	9 (4·9)	36 (14·2)	41 (15·0)	125 (41·8)	91 (45·2)	77 (33·3)	49 (28·3)	119 (31·9)	120 (35·8)
17–18 years	8 (3·6)	3 (1·7)	31 (10·9)	38 (14·6)	94 (34·3)	34 (17·0)	38 (18·2)	16 (10·5)	121 (34·4)	106 (27·6)
19–20 years	4 (2·7)	1 (0·5)	14 (6·0)	12 (4·8)	31 (15·4)	22 (12·2)	21 (12·7)	11 (6·9)	44 (13·8)	31 (9·1)
21–22 years	9 (5·2)	2 (1·1)	11 (5·8)	6 (2·5)	25 (13·9)	3 (1·6)	8 (6·5)	3 (2·2)	14 (7·6)	15 (5·8)
23–24 years	3 (1·9)	6 (2·8)	8 (5·5)	5 (2·7)	22 (12·9)	6 (3·1)	1 (1·2)	3 (2·0)	2 (0·9)	0 (0·0)
Age effect, OR	0·855*	0·576**	0·710***	0·632***	0·464***	0.715**	0.593***	0.584***	0.516***	0.475***
Interaction between gender and age effect, F-stat	1·60		1·13		10·99**		0·02		0·37	
**Intimate partner violence**										
13–14 years	0 (0·0)	1 (0·3)	---	---	2 (0·6)	2 (0·7)	1 (0·3)	4 (1·9)	9 (2·5)	3 (0·7)
15–16 years	1 (0·4)	0 (0·0)	---	---	12 (4·1)	8 (4·0)	1 (0·6)	13 (7·6)	22 (5·8)	10 (3·1)
17–18 years	1 (0·3)	5 (2·7)	---	---	13 (4·7)	29 (14·8)	3 (1·4)	38 (25·6)	23 (6·6)	15 (3·8)
19–20 years	2 (1·4)	5 (3·3)	---	---	27 (13·3)	17 (9·4)	4 (2·4)	27 (16·4)	66 (20·5)	22 (6·4)
21–22 years	3 (2·1)	9 (5·9)	---	---	15 (8·2)	27 (13·3)	7 (5·4)	27 (17·8)	22 (11·4)	24 (9·4)
23–24 years	2 (1·4)	10 (5·0)	---	---	23 (13·5)	21 (11·1)	4 (3·1)	17 (9·8)	14 (8·0)	30 (11·2)
Age effect, OR	1·649***	1·614***	---	---	1·516***	1·350***	1·614***	1·210**	1·285**	1·552***
Interaction between gender and age effect, F-stat	0·02		---		1·45		4·21*		1·85	
**Sexual violence, any perpetrator**										
13–14 years	0 (0·0)	5 (2·2)	31 (9·0)	21 (8·4)	11 (3·4)	23 (9·0)	28 (9·7)	26 (12·1)	10 (2·7)	47 (12·0)
15–16 years	1 (0·5)	8 (4·6)	34 (13·8)	70 (26·3)	11 (3·9)	23 (11·3)	36 (15·7)	50 (28·7)	30 (8·3)	57 (17·1)
17–18 years	1 (0·4)	9 (5·2)	39 (14·0)	61 (24·2)	17 (6·3)	35 (18·2)	25 (12·3)	53 (36·1)	33 (9·7)	72 (19·4)
19–20 years	2 (1·0)	4 (3·1)	50 (22·1)	74 (32·1)	26 (13·4)	23 (12·7)	20 (12·1)	22 (13·3)	59 (18·6)	39 (11·8)
21–22 years	5 (3·2)	6 (4·4)	30 (17·8)	50 (22·5)	13 (7·8)	13 (6·3)	18 (15·0)	16 (10·6)	16 (8·3)	41 (16·9)
23–24 years	0 (0·0)	4 (2·3)	31 (23·2)	48 (24·9)	21 (12·7)	16 (9·0)	10 (8·3)	17 (9·8)	14 (8·2)	30 (11·8)
Age effect, OR	1·475***	0·992	1·225**	1·142**	1·332**	0·95	0·992	0·953	1·181*	0·987
Interaction between gender and age effect, F-stat	8·45**		0·79		10·82**		2·03		3·28	
Sample size	1,255	1,121	1,459	1,457	1,456	1,227	1,132	1,030	1,171	1,968

Note: Age effects are measured using odds ratios and differences in age effects between males and females are assessed using the F-statistic from adjusted Wald tests.

Estimates are significant at *p<0·05, **p<0·01, and ***p<0·001.

Data were weighted to be reflective of the larger population for each country and adjusted for clustering and stratification in the sampling design.

### Physical violence by an adult community member

Similar age effects were observed for exposure to physical violence by a community member. For both males and females, the risk of experiencing this form of violence decreased with age in all countries in the sample. However, in Kenya, the odds of experiencing physical violence by an adult in the community decreased with age significantly more for males than for females (F-statistic = 10·99, p<0·01).

### Intimate partner violence

In contrast to findings pertaining to physical violence, analysis revealed that risk of IPV increased with age for both males and females in all contexts reviewed (Cambodia, Kenya, Malawi, Tanzania). The levels of risk experienced were significantly higher for females in almost every context. This pattern was particularly pronounced in some countries. Additionally, in Malawi, females experienced a significantly greater increase in risk of IPV with age than did males (F = 4.21, p<0.05). For some countries, the highest prevalence of IPV appears to be at ages 17–18 or 18–19, followed by slight decreases.

### Sexual violence

As stated above, females were victimized at higher rates than males in every country in the sample (Table 1). Further, risk of sexual violence also increased with age in many countries in our sample, with different patterns across countries and for females and males. In Kenya and Tanzania, analyses revealed an increasing risk of sexual violence with age for males (Kenya OR = 1.332, p<0.01; Tanzania OR = 1.181, p<0.05), but not for females. The same was true in Cambodia (OR = 1.475, p<0.001), although sexual violence in this context was relatively low for all age bands. In Haiti, however, the odds of experiencing sexual violence increased with age to similar degrees for males and females (F-statistic = 0.79). The prevalence of sexual violence appears to be highest in some countries at ages 17–18 or 18–19, followed by slight decreases.

## Discussion

Overall, findings reveal important patterns in the types of violence experienced at different ages, as well as a few differences between males and females that emerge across the five countries. Analyses demonstrate that risk of experiencing physical violence by an adult caregiver or member of the community decreased with age across all contexts. However, stratified analyses made apparent differences in experiences for males and females in some countries. In Haiti, for example, the odds of physical violence by an adult caregiver decreased significantly with age more for males than for females. These broader patterns make sense conceptually: as adolescents age into young adulthood, they are less likely to live within their parental home [28]. In contrast, risk of IPV increased with age across nearly all contexts for both sexes. Similar results have been found in high-income countries such as the United States, where a nationally representative survey of adolescents ages 12–21 found that risk of IPV increased across three-year age bands for both females and males [29]. However, it is important to note that while these patterns are consistent with age effects, the risk levels are different for each country.

### Tailoring strategies for prevention and starting early

In order to mitigate the negative effects of violence and prevent early exposure, country-, age-, and sex- specific data can offer an important starting point. Identification of groups vulnerable to violence in early adolescence allows stakeholders to effectively target upstream solutions, such as primary–and even secondary and tertiary—prevention programs for younger populations of children. Given the fact that previous research shows that more than 50% of children as young as 2–14 years old have experienced any form of violence in the past year, there is a clear need to target such programs as early in the lifespan as is feasible [30]. A Canadian school-based violence prevention program focused on healthy relationship skills in early adolescence, for example, demonstrated a positive effect on reduction of physical IPV at a 2·5-year follow-up [31]. Similarly, school-based programs focused on improving social and emotional learning faculties amongst elementary school students have demonstrated longitudinal effects on reduction of aggressive attitudes and behavior [32].

The age-, sex- and country-level differences observed in the present analysis highlight why prevention efforts should be altered to more effectively address the specific risks faced by adolescents and young adults. For example, our findings suggest that corporal punishment may be disproportionately affecting boys in some countries, particularly in the early years of adolescence. This finding has been echoed in other recent studies that have found that, where gender differences are observed in experiences of corporal punishment, boys are more likely to report experiencing physical abuse from a parent than are girls [33]. Some parenting programs that target a reduction in physical violence against children and adolescents have been shown to be successful, but strategies may need to be adapted to different phases of child development to maximize impact. For example, the *ACT Raising Safe Kids* and *Parents/Families Matter*! programs, which have been implemented in several LMICs and have been shown to be effective in reducing harsh parenting practices for parents of younger children and adolescents, might need to be tailored to demonstrate the same impact for adolescents over 12 years of age [34, 35]. The *REAL Fathers* program, by incorporating a gender lens and targeting young fathers ages 16–25, has achieved success in preventing both participants’ use of IPV against female partners and physical violence against their 1–3 year-old children. However, the *REAL Fathers* program has yet to be tested with fathers of older children, where a different type of gender lens may need to be applied to account for gender differences in corporal punishment victimization [36]. Programs should usefully look across phases of child development and gender to understand and address how parenting practices differ for boys and girls across the child life span.

In addition to considering programming that is responsive to age and sex differences, it is important to acknowledge local context in such interventions [37]. The West African NGO Tostan recognizes the value of such local adaptation and has successfully leveraged this approach to reduce harmful practices against girls in the region. Specifically, an evaluation of their Community Education Program, which tailors program discussions around local constructions of human rights and harmful practices, found that 77% of graduated communities had permanently eradicated the practice of female genital mutilation a decade after program participation [38]. SASA!, a community mobilization intervention aimed at reducing gender-based inequality and violence in Uganda, also draws heavily on local conceptions of power and inequality; rather than implementing predetermined intervention activities, the program utilizes a bottom-up approach to respond to the specific needs and realities of each community [39]. As a result, evaluations of SASA! suggest that the program not only led to a decline in violence against women, but also reduced children’s exposure to corporal punishment and even led some community members to intervene in potential cases of child abuse [40]. In 2016, key global partners released INSPIRE, a technical package of evidence-based interventions to prevent and respond to violence against children. While INSPIRE has huge potential for policy and advocacy efforts, there is some risk that such a top down approach may privilege interventions shown to be effective in one context, possibly overshadowing effective community-based approaches that are yet unevaluated by large-scale RCTs.

### Deepening our understanding: A need for data on norms alongside information on incidence

Collecting data on descriptive and injunctive norms surrounding violence, alongside data on experiences of violence, may be critical for the development of effective programming. Research on injunctive norms in other contexts has helped to illuminate how harmful norms exacerbate females’ experiences of violence [41]. In particular, data on injunctive norms can help shed light on instances where certain types of violence are especially elevated, normalized, and even accepted for a certain age group or sex. Research on dating violence perpetration in other contexts has found that injunctive norms surrounding acceptability of dating violence and gender role attitudes work synergistically to increase the likelihood of IPV perpetration amongst males [42]. Information on norms surrounding specific types of violence can help to clarify whether and why individuals consider use of that type of violence against a specific age group or sex to be acceptable. Together, data on norms and experiences can contribute to the design of more effective programs, particularly those engaging the broader community.

### Recognizing the multiple roles boys and men hold

Data on violence exposure for 18–24 year olds in the VACS has been largely unexplored. Most analyses of VACS data have focused on childhood violence (before age 18), with data from ages 18–24 used to assess later impacts of childhood violence on health, wellbeing and behavior, and currently-held gender attitudes and norms [15, 43–44]. The analyses in this paper represent the first detailed examination of reports of past-year violence among older adolescents and young adults, and reveal important trends. This analysis may be particularly important in the work to engage men and boys in ending violence, recognizing that they hold multiple identities: survivors of violence themselves, potential champions of change, and the most likely perpetrators of violence against women and girls [15]. Despite growing evidence that violence in childhood (both witnessing and experiencing) is linked to males’ increased risk of perpetration of IPV and non-partner sexual violence later in life, the experiences of young males, particularly young adult males, with violence are often unknown [45–47]. This population may be left out of data on violence against children (which stops at age 18) and gender-based violence data (which primarily focuses on the victimization experiences of women). As research, programs, and policies are developed to better address the interlinkages of violence against children and violence against women, it is increasingly vital to create and use sex- and age-disaggregated lenses.

### Limitations

Findings from this analysis should be considered alongside a few limitations. First, due to the self-reported nature of the outcomes of interest, it is possible that some participants were reluctant to disclose their violence experiences, thereby resulting in underestimations of exposures to different violence types. In order to minimize the likelihood of reporting bias for sensitive information, great care was taken to recruit, select, and train interviewers to build rapport and gain trust with participants. Second, the analysis presented here comprises multiple comparisons of violence risks. Given the ambiguous guidelines relating to which types of adjustments or Bonferroni corrections should be used for such complex comparisons, such adjustments were not made. Finally, due to small sample sizes the age effects may reflect spurious relationships; future research should explore these analyses with larger samples to confirm results.

## Conclusion

Early exposure to violence is associated with the development of negative short- and long-term physical and mental health effects throughout the life course, as well as increased likelihood of future perpetration of violence. While experiences of violence differ significantly throughout adolescence and into young adulthood around the world, one thing remains clear: violence against adolescents and young adults is prevalent and problematic, especially in LMIC. Given that adolescents account for 24% of the global population, and 9 out of 10 adolescents live in less developed countries [1, 2], it is essential that we work towards identifying when, why, and how they experience violence. Ultimately, our analysis demonstrates the importance of conducting national violence studies, and of evaluating differences across age and by sex when doing so in order to identify the nuanced experiences of violence for adolescents and young adults in order to generate impactful policies and programs targeting violence against youth.

## Abbreviations

IPV: Intimate partner violence
LMIC: Low and middle-income countries
VACS: Violence Against Children Surveys
